# Preparation and Recovery Behavior of Lithium Chloride (LiCl) from Lithium Iron Phosphate (LiFePO_4_) Cathode Active Materials via Hydrogen Reduction and CaCl_2_-Assisted Thermal Chlorination

**DOI:** 10.3390/ma19071474

**Published:** 2026-04-07

**Authors:** Tae-Jun Jeon, Jei-Pil Wang

**Affiliations:** Department of Metallurgical Engineering, BB21 Plus Team, Pukyong National University, Busan 48513, Republic of Korea

**Keywords:** LiFePO_4_ (LFP), hydrogen reduction, chlorination roasting, CaCl_2_-assisted chlorination, water leaching

## Abstract

In this study, lithium was recovered from LiFePO_4_ (LFP) cathode active materials through a two-step thermal process combining hydrogen reduction and chlorination roasting. Hydrogen reduction was conducted while varying temperature and holding time to promote oxygen removal from LFP and induce phase separation into Li_3_PO_4_ and iron phosphides (FeP and Fe_2_P). Based on stoichiometric assessment using the degree of LFP decomposition and the reduction in oxygen moles, the optimal hydrogen-reduction condition was determined to be 900 °C for 1 h. Subsequently, CaCl_2_ was selected as an appropriate chlorination agent using thermodynamic considerations, and the hydrogen-reduced product was reacted with CaCl_2_ to convert Li_3_PO_4_ into water-soluble LiCl. The mass of LiCl produced was quantified as a function of reaction temperature. Water leaching enabled the separation of LiCl from the insoluble residues, resulting in an overall lithium recovery of 71.7%.

## 1. Introduction

Lithium iron phosphate (LiFePO_4_, LFP) cathodes are being rapidly adopted in electric vehicles (EVs) and energy storage systems (ESSs) due to their cobalt-free composition, high thermal stability, long cycle life, and comparatively low cost. As the global EV battery market expands, LFP penetration has reached a substantial level; the International Energy Agency (IEA) reported that LFP accounted for nearly half of the global EV battery market in 2024 [1]. This increasing deployment will inevitably lead to large-scale generation of end-of-life batteries, making environmentally responsible treatment of spent LFP batteries and the recovery of metal resources—particularly lithium—an urgent challenge from both environmental and resource security perspectives. Moreover, considering the rising lithium demand and supply-chain concentration, it is strategically important to recover lithium from spent LFP batteries in reusable chemical forms (e.g., LiCl, Li_2_CO_3_, or LiOH) that can be reintegrated into battery-material supply chains as part of a circular economy [2].

Research on LFP recycling has broadly evolved along three routes: (i) direct regeneration, (ii) hydrometallurgy, and (iii) thermal/pyrometallurgical routes involving decomposition followed by selective recovery. Zhao et al. summarized recent progress in direct regeneration approaches (e.g., relithiation and structural restoration), noting that while process simplification is attractive, practical implementation is hindered by variability in feed condition, impurity content, and degradation degree, as well as scale-up challenges [3]. In parallel, Barbosa de Mattos et al. and Visone et al. critically reviewed diverse LFP recycling pathways encompassing both hydrometallurgical routes (acid leaching–solvent extraction/precipitation) and hybrid/thermal routes (thermal treatment–phase control–leaching) [2,4]. They emphasized that conventional hydrometallurgical processes can achieve high lithium recovery but may suffer from intensive reagent consumption, wastewater management, and increased flowsheet complexity. Accordingly, salt-assisted roasting followed by water leaching has been actively investigated for LFP recycling [5,6,7,8]. More recently, chlorination roasting has been proposed as an alternative strategy to obtain highly water-soluble lithium salts. Kim et al. highlighted that converting lithium into chloride species using chlorine donors can be advantageous for subsequent water leaching and solid–liquid separation [9]; similar chlorination-roasting approaches have also been reported for other spent LIB cathode chemistries [10,11]. In particular, Cao et al. reported CaCl_2_-assisted chlorination roasting of LFP. They discussed the formation behavior of phosphate-containing byproducts (e.g., calcium phosphate/chlorophosphate/apatite-like phases), emphasizing that reaction-condition control is critical to suppress side reactions and maximize lithium recovery [12,13].

Although hydrometallurgical routes often deliver high lithium recovery, the multi-step leaching and purification stages can be constrained by reagent consumption and the burden of wastewater/sludge treatment, which may limit techno-economic feasibility and environmental performance [4]. Direct regeneration can reduce process steps, yet stable product quality remains challenging for industrialization because cathode performance after regeneration strongly depends on feed variability and impurity carryover [3]. CaCl_2_-based chlorination roasting offers the benefit of converting lithium into water-soluble LiCl, enabling rapid separation by water leaching and solid–liquid separation. However, it has been reported that phosphate-derived phases formed during reaction—such as calcium phosphate/chlorophosphate/apatite-like compounds—may immobilize or entrap LiCl (e.g., via absorption/occlusion of molten LiCl or incorporation into solid byproducts). In addition, chloride ions can be consumed by byproduct formation, thereby decreasing the practical LiCl yield relative to the theoretical production [7]. Therefore, a remaining technical gap is to control phosphate chemistry during chlorination by isolating and concentrating lithium into a reactive target phase prior to chlorination, rather than chlorinating the intact LFP matrix, where phosphate-derived byproducts can consume chloride and/or trap LiCl.

To address this gap, the present study proposes a two-stage thermal route to selectively and efficiently convert lithium from LFP cathode active materials into LiCl. First, hydrogen reduction is employed to remove oxygen-containing components and induce lithium into a lithium phosphate phase (Li_3_PO_4_), while simultaneously separating iron into phosphide phases (Fe_x_P), thereby enabling phase-separation-based pretreatment [14]. Second, the separated Li_3_PO_4_ is converted into LiCl via CaCl_2_-assisted thermal chlorination [15,16,17]. The generated LiCl is then recovered through water leaching and vacuum filtration to separate soluble LiCl from insoluble residues, and residual Ca^2+^ in solution is removed using an ion-exchange resin to improve LiCl purity. This approach simplifies the reaction system through “formation of a lithium target phase (Li_3_PO_4_) → selective chlorination,” thereby mitigating phosphate byproduct formation and LiCl entrapment that are frequently discussed for direct CaCl_2_ chlorination roasting of LFP [9,18]. Recovered LiCl was targeted because it is highly water-soluble, enabling efficient separation from insoluble phosphate/iron-bearing phases by simple water leaching and solid–liquid separation, and because it serves as a practical lithium intermediate that can be converted to downstream lithium chemicals (e.g., Li_2_CO_3_ via carbonation/precipitation and LiOH via causticization or electrochemical routes). Accordingly, this route aims to recover lithium in a chemically reusable form that is readily compatible with downstream lithium-salt production and circular supply chains.

In contrast to direct CaCl_2_ chlorination of LFP—where phosphate-derived byproducts can consume chloride and/or physically retain molten LiCl—this work introduces an original two-stage thermal design that decouples lithium liberation from chlorination. Hydrogen reduction first converts LiFePO_4_ into a lithium-rich target phase (Li_3_PO_4_) while partitioning iron into phosphides (FeP/Fe_2_P), thereby simplifying the subsequent chlorination chemistry and enabling selective conversion of the lithium-bearing phase to water-soluble LiCl. This phase-separation-first strategy provides a clear mechanistic advantage: it reduces parasitic reactions associated with the intact phosphate matrix and focuses on chlorinating the lithium-bearing phase, thereby improving the controllability and reproducibility of LiCl formation. In addition, we introduce an aqueous purification step—Ca^2+^ removal by strong-acid cation exchange—to reduce calcium impurities originating from CaCl_2_ and Ca–phosphate equilibria, improving the chemical purity of recovered LiCl without introducing additional alkali ions or requiring precipitation reagents that may generate secondary waste solids. Together, these features define the scientific novelty and technological originality of the proposed route relative to previously reported LFP recycling approaches.

## 2. Materials and Methods

### 2.1. Overview of Process Flow

**Figure 1:**
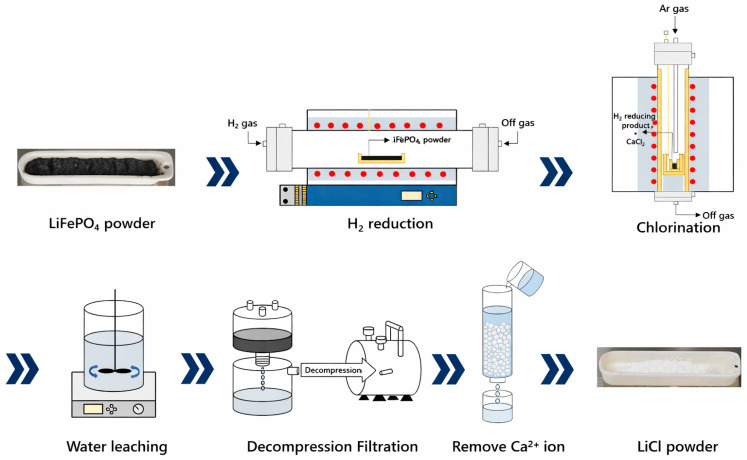
Overall process scheme for lithium recovery from LiFePO_4_ cathode powder via hydrogen reduction, CaCl_2_-assisted chlorination roasting, water leaching/vacuum filtration, and Ca^2+^ removal by ion exchange, including photographs of the starting LiFePO_4_ powder and the final recovered LiCl powder prepared in this study. Blue arrows indicate the process flow direction, and red circles denote the heating zone.

In this study, we investigated a two-step thermal strategy to recover lithium from LiFePO_4_ as water-soluble LiCl. The study was designed around three questions: (i) under what hydrogen-reduction conditions does LiFePO_4_ fully decompose and lithium partition into Li_3_PO_4_ while iron segregates into phosphides; (ii) under what chlorination conditions is Li_3_PO_4_ most effectively converted into LiCl while minimizing lithium loss to phosphate-derived solids; and (iii) how effectively can LiCl be separated and purified from calcium-containing impurities. Accordingly, the progress of each step was evaluated by phase identification (XRD), composition measurements (ICP–OES), and a lithium mass balance to quantify overall recovery. Optimal conditions were determined using explicit criteria: disappearance/appearance of characteristic phases in XRD, agreement between experimental and theoretical mass change during hydrogen reduction, lithium content in the leachate-derived product, and reduced Ca content after ion-exchange purification. The overall experimental procedure is summarized in Figure 1. First, a hydrogen-reduction experiment was conducted to promote the reaction between LiFePO_4_ powder and hydrogen at elevated temperature, thereby removing oxygen from LiFePO_4_ as water vapor (H_2_O (g)). This step simultaneously converts the lithium-bearing phase into lithium phosphate (Li_3_PO_4_) and induces phase separation of the iron-bearing component into iron phosphide phases.

The overall experimental procedure is summarized in Figure 1. Unless otherwise stated, all gas flow rates were controlled at 300 cm^3^ min^−1^, and the heating rate was fixed at 5 °C min^−1^. For hydrogen reduction, 10.0 g of LiFePO_4_ powder was loaded into a boat-type crucible and treated in a horizontal tube furnace under H_2_ flow at 700–900 °C for 1–3 h to induce phase separation into Li_3_PO_4_ and Fe phosphides. The optimal reduction condition was determined to be 900 °C for 1 h. For chlorination, the hydrogen-reduced product (7.292 g from 10.0 g feed under the optimal reduction condition) was mixed with CaCl_2_ according to the stoichiometric ratio of Equation (2) (2Li_3_PO_4_:3CaCl_2_; corresponding CaCl_2_ mass = 3.562 g for the present batch) and reacted in a vertical tube furnace at 700–1000 °C for 3 h in a magnesia crucible. Water leaching was performed using deionized water at a solid-to-liquid ratio of 1:10, under stirring at 300 rpm and 25 °C for 24 h, followed by vacuum filtration to separate insoluble residues from the LiCl-containing filtrate. Residual Ca^2+^ in the filtrate was removed by adding 1.56 g of a strong-acid cation-exchange resin (H-form) and stirring at 300 rpm for 1 h, after which the resin was separated by filtration. The purified LiCl solution was dried at 120 °C for 24 h, followed by dehydration at 400 °C for 5 h to obtain anhydrous LiCl powder. No chemical leaching agents (acids/alkalis) were used; distilled/deionized water without additives was employed as the lixiviant.

Subsequently, a chlorination experiment was performed to produce lithium chloride (LiCl) by reacting Li_3_PO_4_ with calcium chloride (CaCl_2_). Because LiCl is highly soluble in water, the generated LiCl was separated and recovered by water leaching, followed by solid–liquid separation. The hydrogen-reduction step proceeds according to Equation (1), while the chlorination step proceeds according to Equation (2).

3LiFePO_4_ + 8H_2_ (g) → Li_3_PO_4_ + FeP + Fe_2_P + 8H_2_O (g) (1)

2Li_3_PO_4_ + 3CaCl_2_ → 6LiCl + Ca_3_(PO_4_)_2_ (2)

### 2.2. Sample Characterization and Pretreatment

**Figure 2:**
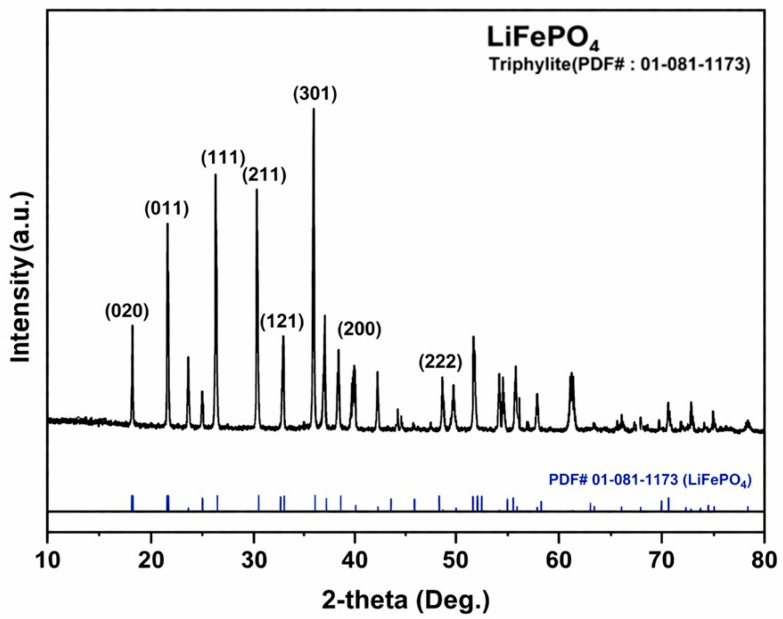
X-ray diffraction (XRD) pattern of the as-received LiFePO_4_ (LFP) powder (reference: triphylite, PDF #01-081-1173).

This study aims to recover lithium from LiFePO_4_ (LFP) powder in the form of lithium chloride (LiCl). To confirm the phase identity and elemental composition of the starting material, the as-received LFP powder was characterized by X-ray diffraction (XRD) and bulk chemical analysis by Inductively Coupled Plasma–Optical Emission Spectrometry (ICP-OES), as shown in Figure 2 and Table 1. The measured composition was in close agreement with the theoretical values, indicating that the feed material is chemically consistent with nominal LiFePO_4_ stoichiometry.

**Figure 3:**
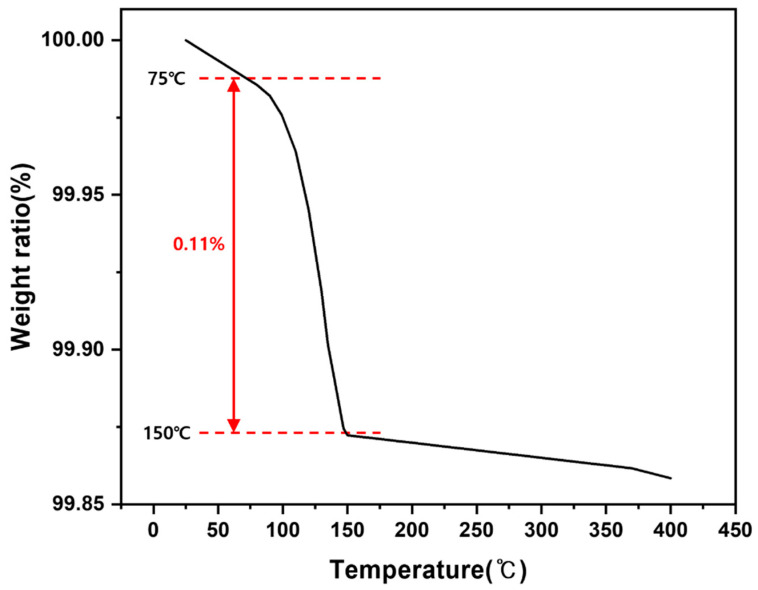
Thermogravimetric analysis (TGA) curve of the as-received LiFePO_4_ powder measured under Ar atmosphere.

The XRD pattern confirms that the starting powder is single-phase LiFePO_4_. Because moisture and adventitious contaminants can be adsorbed on the powder surface during storage and may affect subsequent high-temperature reactions, a mild thermal pretreatment was applied to minimize their influence. To determine an appropriate pretreatment temperature, thermogravimetric analysis (TGA) of the as-received LFP powder was conducted under an Ar atmosphere at a heating rate of 5 °C min^−1^ over 25–400 °C, as shown in Figure 3. A small mass loss was observed and attributed primarily to the removal of trace moisture and/or volatile species. The most pronounced mass decrease occurred between approximately 100 and 200 °C, suggesting that dehydration/desorption is significant within this temperature range.

**Figure 4:**
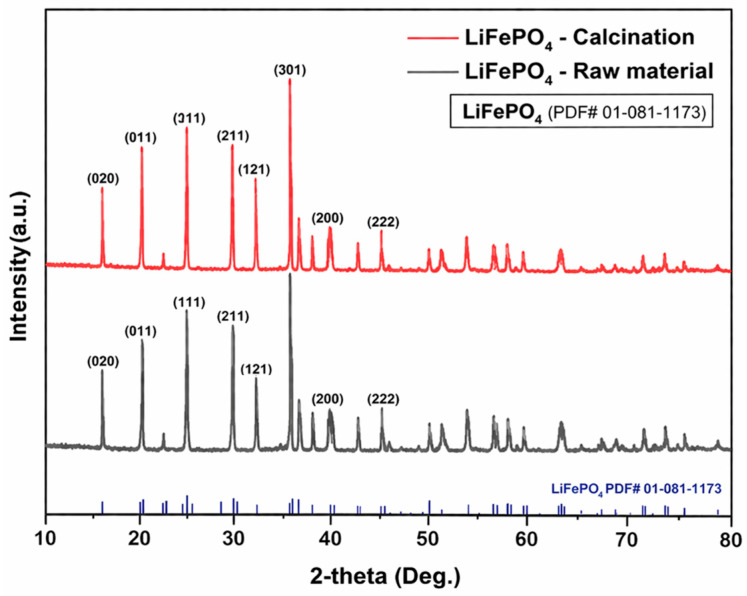
Comparison of XRD patterns between raw LiFePO_4_ powder and calcined LiFePO_4_ powder.

Based on the TGA result, the LFP powder was calcined under Ar at 150 °C for 3 h, and the phase stability after pretreatment was verified by XRD. As shown in Figure 4, the XRD patterns of the calcined and raw samples are essentially identical, indicating that no detectable phase transformation occurred during the pretreatment. Therefore, the LFP powder pretreated at 150 °C for 3 h under Ar was used as the feed material for subsequent experiments. Elemental analysis further confirmed the effectiveness of the pretreatment: the hydrogen content decreased from 0.017 wt.% in the raw powder to below the detection limit after calcination (Table 2). SEM/TEM characterization was not included because representative intermediate and final solids were not retained after completion of the experiments. Instead, phase and compositional evolution before and after each processing step was verified by sequential XRD and ICP–OES analyses, complemented by mass-balance assessment.

### 2.3. Experimental Apparatus

Three main types of equipment were used in this study: (i) a horizontal tube furnace, (ii) a vertical tube furnace, and (iii) a water-leaching and vacuum-filtration setup.

#### 2.3.1. Horizontal Tube Furnace (For Drying and H_2_ Reduction)

**Figure 5:**
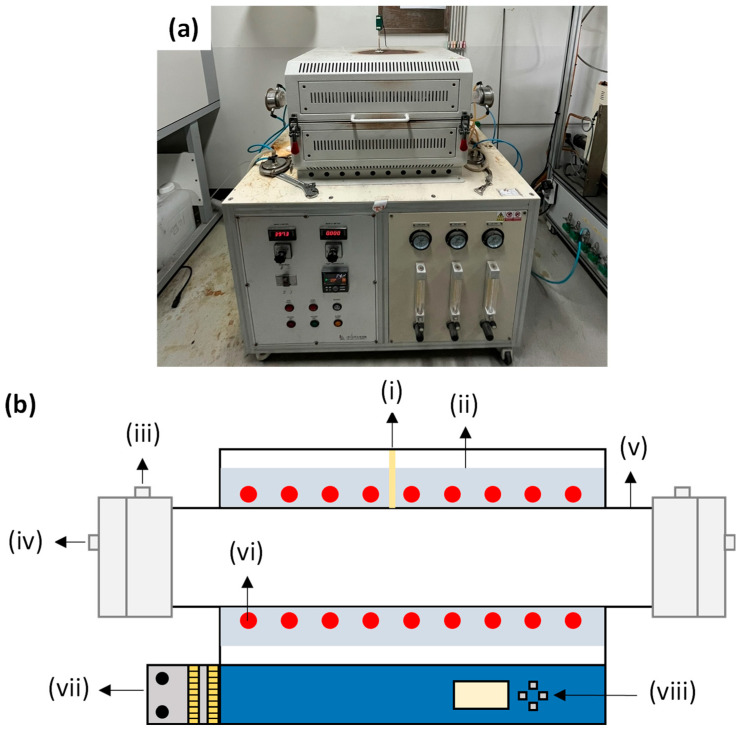
Horizontal tube furnace used for drying, H_2_ reduction, and dehydration of the LiCl-containing product: (**a**) photograph of the furnace system; (**b**) schematic diagram showing the main components: (i) thermocouple, (ii) alumina refractories, (iii) water-cooling line, (iv) gas line, (v) quartz tube, (vi) SiC heater, (vii) flowmeter, and (viii) controller.

A horizontal tube furnace was employed for (i) removing moisture adsorbed on the feed powder, (ii) conducting the H_2_ reduction to convert lithium in LiFePO_4_ to Li_3_PO_4_ while promoting phase separation of the Fe-bearing component, and (iii) drying the final LiCl-containing filtrate and removing hydration water from the recovered product. The furnace was operated over 25–1050 °C. A schematic of the horizontal tube furnace and its main components is shown in Figure 5.

Temperature was monitored using a thermocouple and controlled via an external controller/display, allowing precise adjustment of the heating program (heating rate), holding time, and target temperature. Heating was provided by SiC heating elements, which generate heat by electrical resistance. To minimize thermal deformation and maintain stable operation at high temperatures, the furnace was equipped with alumina refractories and a water-cooling line. Process gas flow was regulated using a flowmeter and introduced through the gas line into a quartz tube, in which the sample crucible was placed for reaction. When using the horizontal tube furnace, a boat-type crucible was adopted to maximize the exposed surface area and facilitate gas–solid reaction.

#### 2.3.2. Vertical Tube Furnace (For Chlorination)

**Figure 6:**
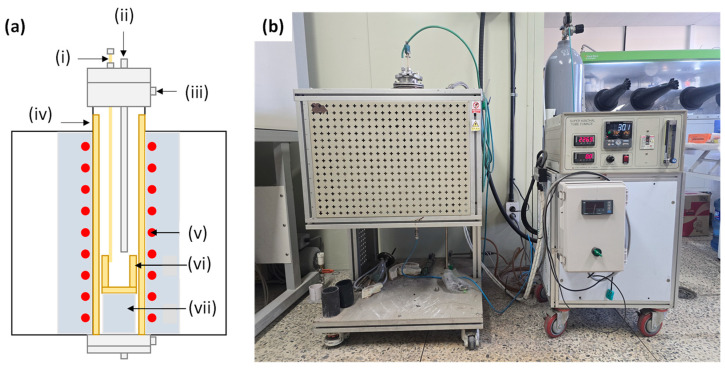
Vertical tube furnace used for CaCl_2_-assisted chlorination roasting: (**a**) schematic diagram of the vertical tube furnace; (**b**) photograph of the experimental setup. The main components are: (i) thermocouple, (ii) gas line, (iii) water-cooling line, (iv) alumina tube, (v) Kanthal heater, (vi) protection crucible, and (vii) alumina refractories.

A vertical tube furnace was used for the chlorination step, in which Li_3_PO_4_ reacts with CaCl_2_ at high temperature to produce LiCl through melt-mediated reaction under near-equilibrium conditions. The vertical furnace enables stable operation at elevated temperatures and was used over the 25–1700 °C range. A schematic of the vertical tube furnace and its main components is provided in Figure 6.

Similar to the horizontal furnace, the heating program (heating rate), reaction temperature, and holding time were controlled using an external controller. Heat was supplied by a Kanthal heating element, providing a stable thermal environment for reaction between the sample components. The sample was placed in a protection crucible inside an alumina tube, with additional alumina refractories and water cooling to ensure safe and stable high-temperature operation.

#### 2.3.3. Crucible Selection for H_2_ Reduction

**Figure 7:**
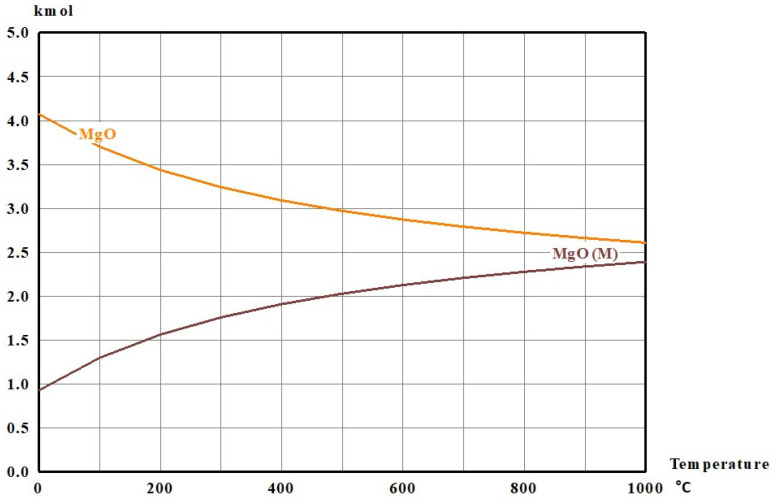
Equilibrium composition calculated using HSC Chemistry 6.0 (Outotec Research Oy, Pori, Finland) for the system 10H_2_ (g) + 5MgO + Li_3_PO_4_ + FeP + Fe_2_P as a function of temperature, showing negligible MgO consumption.

**Figure 8:**
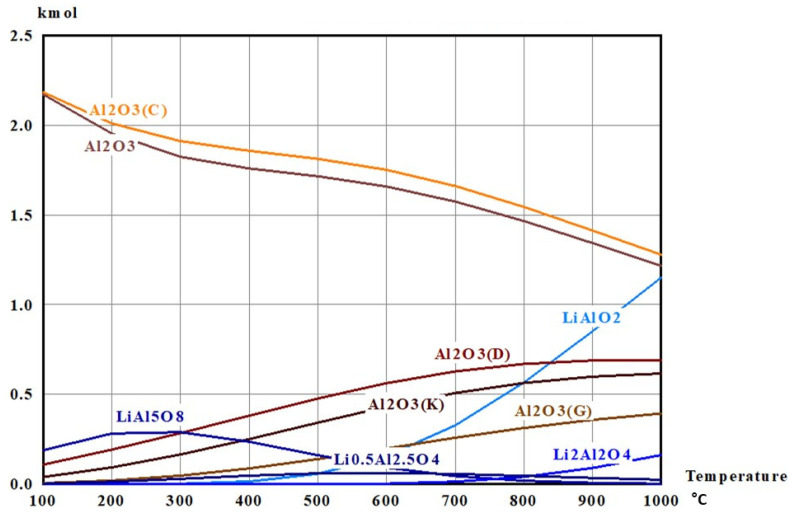
Equilibrium composition calculated using HSC Chemistry 6.0 (Outotec Research Oy, Pori, Finland) for the system 10H_2_ (g) + 5Al_2_O_3_ + Li_3_PO_4_ + FeP + Fe_2_P as a function of temperature, showing alumina consumption and the formation of Li–Al–O and Fe–Al–O phases.

To select a crucible material that remains chemically inert during the hydrogen-reduction treatment, thermodynamic equilibrium calculations were carried out using HSC Chemistry 6 (Equilibrium Composition module). This determines the stable phase assemblage by Gibbs free-energy minimization. Alumina (Al_2_O_3_) and magnesia (MgO) were evaluated as candidate crucible materials because of their widespread use in high-temperature processing and their high thermal and chemical stability. Because LiFePO_4_ is not available in the HSC database, the calculation was performed using the expected solid products after hydrogen reduction, i.e., Li_3_PO_4_, FeP, and Fe_2_P, each set to 1 mol. To represent a hydrogen-rich reducing atmosphere, 10 mol of H_2_(g) was included, which is an excess amount relative to the minimum stoichiometric requirement (8 mol) implied by Equation (1). To assess potential interactions between the reaction mixture and crucible materials, either 5 mol of MgO (MgO crucible case) or 5 mol of Al_2_O_3_ (Al_2_O_3_ crucible case) was added to the reactant set. Equilibrium phase distributions were calculated as a function of temperature over 300–900 °C. The resulting equilibrium compositions are presented in Figure 7 and Figure 8 and Table 2 and Table 3.

In the MgO-containing system, the equilibrium results indicated temperature-dependent redistribution among MgO polymorphs; however, the total amount of MgO remained constant at 5.0000 mol throughout the investigated temperature range (Table 3). This conservation of total MgO suggests that MgO is not chemically consumed and is therefore thermodynamically compatible with the reaction mixture under hydrogen-reduction conditions. In contrast, in the Al_2_O_3_-containing system, the total amount of Al_2_O_3_ was consistently below 5.0000 mol at all temperatures (Table 4), indicating that alumina participates in side reactions with lithium- and/or iron-bearing species. This trend is consistent with the formation of secondary Li–Al–O phases (LixAlyOz) and Fe–Al–O compounds, as predicted by the equilibrium calculations (Figure 8), which may lead to crucible degradation and contamination of the reaction products. Based on these thermodynamic predictions, a magnesia crucible was selected for the hydrogen-reduction experiments.

#### 2.3.4. Water Leaching, Ca^2+^ Removal by Cation-Exchange Resin, and Dehydration of LiCl Hydrate

**Figure 9:**
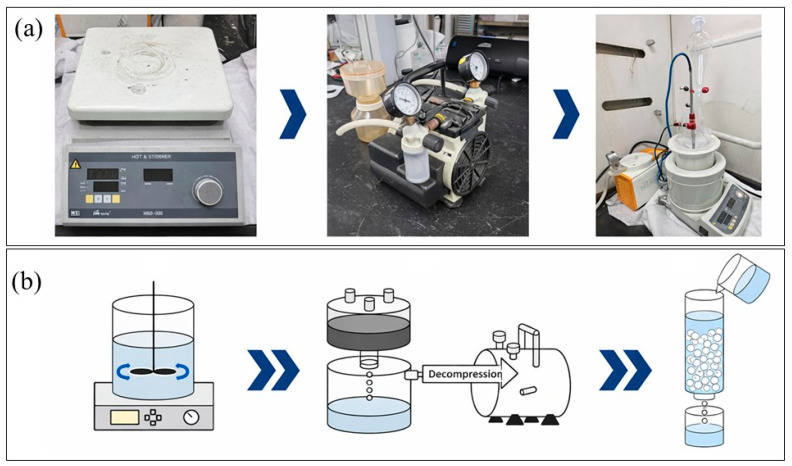
Water-leaching and purification setup for LiCl recovery: (**a**) photographs of the magnetic stirrer/hotplate, vacuum filtration unit with vacuum pump, and resin-based Ca^2+^ removal setup; (**b**) schematic illustration of the overall sequence (water leaching → vacuum filtration → Ca^2+^ removal using a cation-exchange resin). Arrows indicate the process flow direction, and the white circles represent resin beads in the cation-exchange column.

A schematic of the water-leaching, vacuum-filtration, and Ca^2+^ removal procedure is shown in Figure 9. After chlorination, water-soluble LiCl was separated from insoluble phases by water leaching followed by vacuum filtration. The leachate was subsequently purified by removing residual Ca^2+^ using a strong-acid cation-exchange resin (H-form).

LiCl exhibits high solubility in water; as summarized in Table 5, the solubility is approximately 84.25 g per 100 mL at 25 °C. In contrast, the major byproduct expected from chlorination, calcium phosphate (Ca_3_(PO_4_)_2_), as well as iron phosphides (FeP and Fe_2_P) originating from the H_2_-reduction product, are practically insoluble in water. Therefore, solid–liquid separation based on solubility contrast allows LiCl to be recovered into the filtrate, while Ca_3_(PO_4_)_2_/FeP/Fe_2_P remain in the filter cake. Distilled water was used as the lixiviant, and a solid-to-liquid ratio of 1:10 (g mL^−1^) was adopted to ensure sufficient dissolution. Leaching was conducted using a magnetic stirrer at 300 rpm for 24 h, followed by vacuum filtration to obtain the LiCl-rich solution.

**Figure 10:**
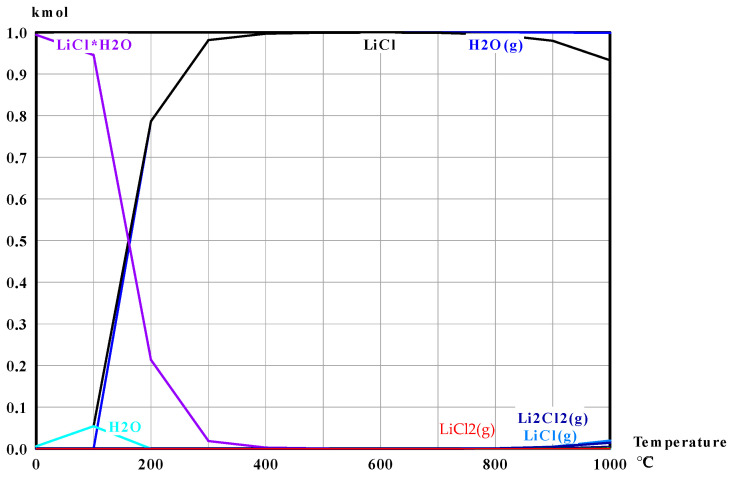
Thermal decomposition and phase evolution of LiCl·H_2_O from 0 to 1000 °C, calculated using HSC Chemistry 6.0 (Outotec Research Oy, Pori, Finland). The red line denotes Li_2_Cl_2_ (g), and the blue line denotes LiCl (g).

Residual Ca^2+^ in the filtrate, originating from CaCl_2_ and calcium-bearing solids, was removed using a strong-acid cation-exchange resin, TRILITE^®^ MCN-116K (H-form, gel type; SAMYANG TRILITE, Seoul, Republic of Korea). Strong-acid cation exchangers typically consist of a styrene–divinylbenzene copolymer matrix functionalized with sulfonic acid groups. In the H-form, divalent and trivalent cations preferentially exchange with H^+^ sites, showing higher selectivity for ions of higher valence and atomic number (e.g., Li^+^ < Na^+^ < Ca^2+^ < Al^3+^ < Th^4+^). The ion-exchange resin was employed to remove dissolved Ca^2+^ remaining in the LiCl-containing filtrate after water leaching and filtration; without this step, Ca^2+^ would remain in the dried product as an impurity (e.g., residual CaCl_2_ or Ca-containing salts), which is undesirable for downstream lithium chemical synthesis and for evaluating LiCl product purity. According to the supplier specification, the resin has a density of 815 g L^−1^ and a total exchange capacity of ≥2.4 eq L^−1^, corresponding to a theoretical Ca^2+^ uptake of approximately 1.2 mol Ca^2+^ per liter of resin (≈48 g Ca). A strong-acid cation-exchange resin (H-form, gel type) was selected because (i) it provides high exchange capacity and strong affinity toward divalent cations such as Ca^2+^ in chloride-rich solutions, (ii) the H-form avoids introducing additional alkali ions (Na^+^/K^+^) into the product stream, and (iii) it enables purification without adding extra precipitants that may generate additional waste solids or induce lithium loss via coprecipitation. In the present work, because the experimental scale was small, a fixed-bed column was not employed. Instead, the Ca concentration in the filtrate was first determined, and the required resin dosage was calculated based on the exchange capacity. The calculated amount of resin was added directly to the LiCl solution and mixed to remove Ca^2+^, after which the resin was separated by vacuum filtration to recover the purified LiCl solution. The purified LiCl solution was dried to obtain solid LiCl, and the lithium recovery was evaluated by comparing the lithium content in the initial LiFePO_4_ feed with that in the final LiCl product. Because LiCl is highly hygroscopic, it can readily form hydrates (LiCl·H_2_O) during ambient drying. To establish suitable conditions for producing anhydrous LiCl while minimizing LiCl loss by volatilization, the thermal decomposition behavior of LiCl·H_2_O was assessed using HSC Chemistry as shown in Figure 10 and Table 6. The calculation indicates that dehydration becomes effective from approximately 300 °C, where water is released predominantly as H_2_O(g). At temperatures above approximately 600 °C, a small fraction of LiCl begins to volatilize. Accordingly, a two-step drying protocol was adopted: (i) primary drying at 120 °C for 24 h to remove free water, followed by (ii) secondary heat treatment at 400 °C for 5 h to remove crystallization water from LiCl hydrates while avoiding significant LiCl volatilization.

## 3. Results and Discussion

### 3.1. Hydrogen Reduction of LiFePO_4_

The purpose of this section is to determine hydrogen-reduction conditions that achieve complete decomposition of LiFePO_4_ and selective partitioning of lithium into Li_3_PO_4_, which is required for subsequent selective chlorination.

#### 3.1.1. Effect of Temperature Under H_2_ Atmosphere

**Figure 11:**
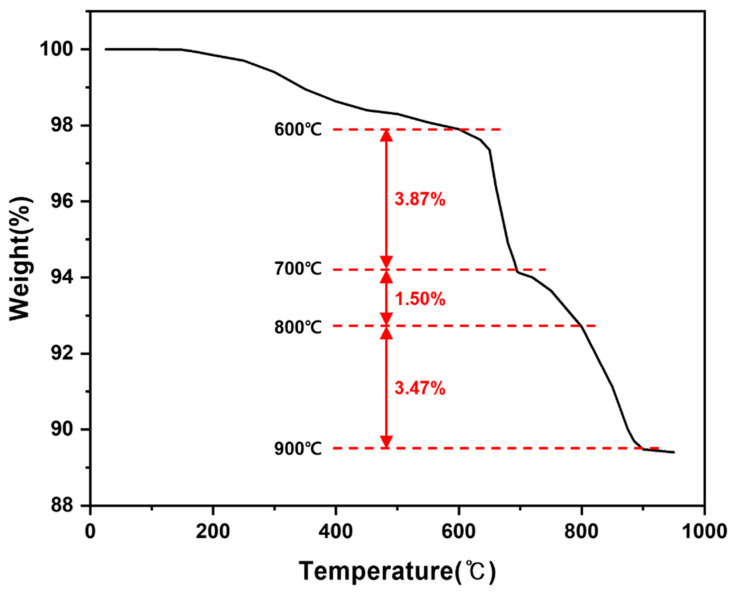
Thermogravimetric response of LiFePO_4_ under H_2_ atmosphere during stepwise heating (100–950 °C; 50 °C increments; 30 min hold at each step; 5 °C min^−1^), indicating a pronounced mass loss region between 600 and 900 °C.

Prior to isothermal hydrogen-reduction experiments, the thermal behavior of LiFePO_4_ powder under a hydrogen atmosphere was examined by monitoring mass changes as a function of temperature, as shown in Figure 11. The sample was heated from 100 to 950 °C at 50 °C intervals, with a holding time of 30 min at each step and a heating rate of 5 °C min^−1^ under H_2_. A pronounced mass decrease was observed primarily between 600 and 900 °C, which is attributed to oxygen removal from the LiFePO_4_ lattice via reaction with H_2_ to form H_2_O(g). Based on this screening result, the isothermal reduction temperatures were set to 700, 800, and 900 °C to identify an optimal condition for complete decomposition and phase separation.

#### 3.1.2. Isothermal Reduction Design and Mass-Change Behavior

Hydrogen reduction of LiFePO_4_ is described by Equation (3), where oxygen is removed as water vapor and lithium is converted to Li_3_PO_4_ while iron forms phosphide phases (FeP/Fe_2_P).

3LiFePO_4_ + 8H_2_ (g) → Li_3_PO_4_ + FeP + Fe_2_P + 8H_2_O (g) (3)

Isothermal experiments were conducted at 700, 800, and 900 °C with holding times of 1, 2, and 3 h, respectively. The heating rate was fixed at 5 °C min^−1^, and the H_2_ flow rate was maintained at 300 cm^3^ min^−1^. The initial sample mass was 10.0 g for all runs. The experimental matrix and resulting mass-retention ratios are summarized in Table 7.

**Figure 12:**
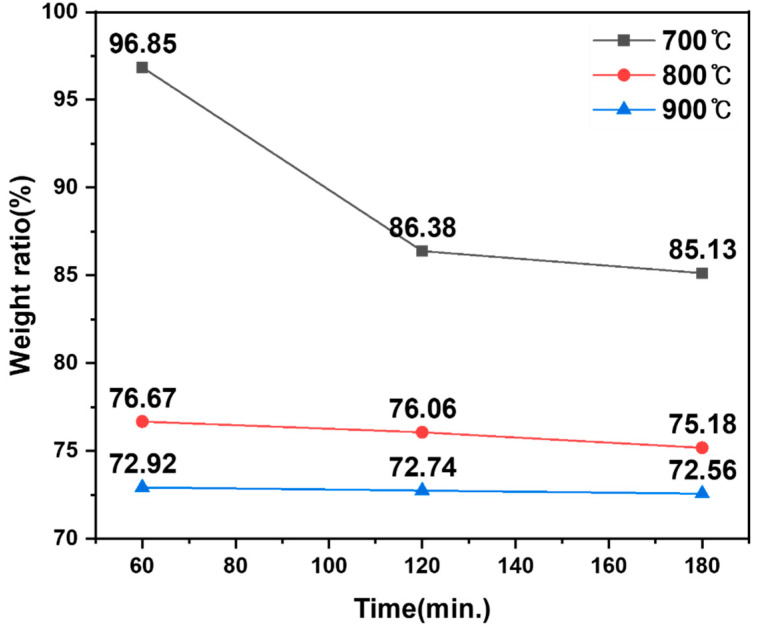
Mass-retention ratio of hydrogen-reduced products as a function of reaction time at 700–900 °C.

The mass-retention ratio decreased with increasing temperature and time, approaching a limiting value at higher temperatures, as shown in Figure 12. In particular, the mass-retention ratio at 900 °C converged near ~72–73%, suggesting that oxygen removal and phase evolution were largely completed within the tested duration.

#### 3.1.3. Phase Evolution Confirmed by XRD

**Figure 13:**
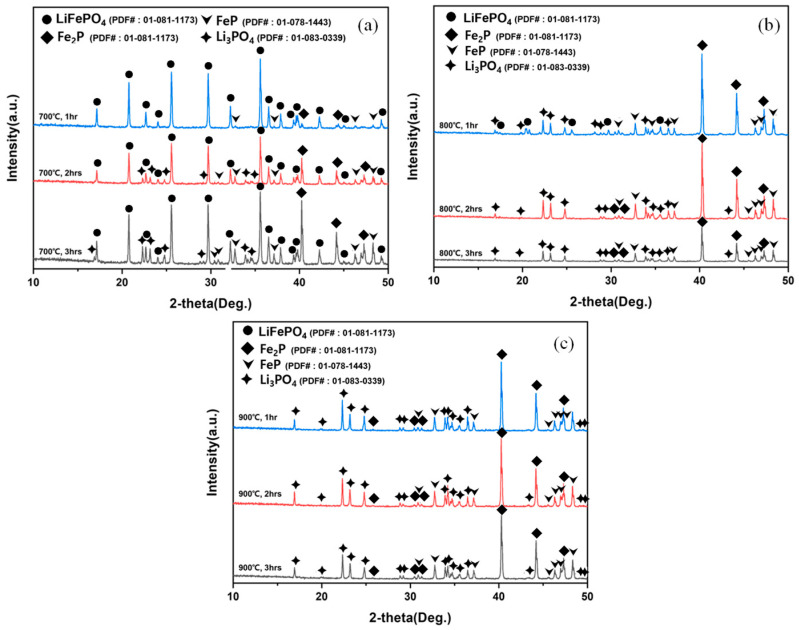
XRD patterns of hydrogen-reduction products obtained at (**a**) 700 °C, (**b**) 800 °C, and (**c**) 900 °C for 1–3 h, showing the progressive disappearance of LiFePO_4_ peaks and the formation of Li_3_PO_4_ and iron phosphides (FeP/Fe_2_P).

XRD patterns of the reduction products obtained at each temperature (700–900 °C) for 1–3 h are compiled in Figure 13a–c. At 700 °C, Li_3_PO_4_ reflections began to appear after 2 h, indicating the onset of phase separation accompanied by oxygen removal. At 800 °C, LiFePO_4_ peaks were no longer detectable from 2 h, implying near-complete decomposition of the starting phase and formation of Li_3_PO_4_ and iron phosphides. From 800 °C (3 h) to 900 °C (3 h), no further changes in the set of detectable phases were observed, suggesting that the principal phase transformation had been completed.

#### 3.1.4. XRD Stoichiometric Validation of Theoretical Mass Change

To determine the optimal reduction condition quantitatively, the theoretical mass change associated with oxygen removal was calculated based on Equation (3) using the stoichiometry of LiFePO_4_ and the amount of oxygen expected to be removed as H_2_O(g). The calculation (basis: 10.0 g LiFePO_4_) predicts a theoretical product mass of approximately 7.296 g, corresponding to a mass-retention ratio of ~72.96%. The calculation summary is provided in Table 8.

#### 3.1.5. Selection of the Optimal Reduction Condition and Product Verification

**Figure 14:**
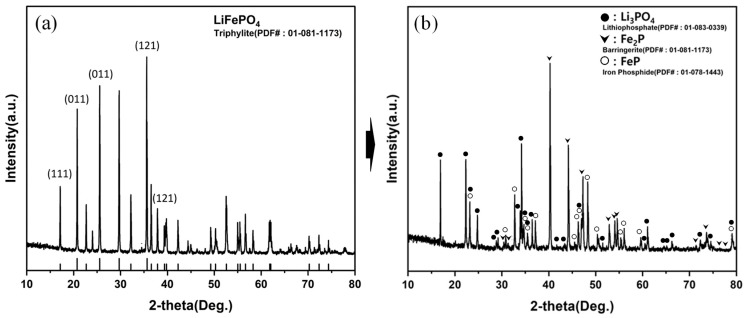
Phase evolution of LiFePO_4_ through hydrogen reduction, illustrated by XRD patterns of the raw material (**a**) and the reduced product (**b**) obtained under the selected condition (900 °C, 1 h), confirming the formation of Li_3_PO_4_ and iron phosphides (FeP/Fe_2_P).

Among the tested conditions, the experimental mass-retention ratio at 900 °C for 1 h (72.92%) was the closest to the theoretical value (~72.96%), and XRD confirmed the absence of LiFePO_4_ peaks with the presence of Li_3_PO_4_ and iron phosphides. The optimal hydrogen-reduction condition was defined as the minimum time/temperature at which the LiFePO_4_ peaks disappeared and the experimental mass-retention ratio agreed with the theoretical value for oxygen removal. Therefore, 900 °C for 1 h was selected as the optimal hydrogen-reduction condition. The chemical composition of the reduced product obtained under the selected condition is summarized in Table 9, where the measured elemental composition is in reasonable agreement with the theoretical values derived from stoichiometry. The phase conversion of Li-containing species from LiFePO_4_ to Li_3_PO_4_ after hydrogen reduction is illustrated in Figure 14.

### 3.2. Chlorination and Water Leaching for LiCl Production

#### 3.2.1. Thermodynamic Feasibility of Chlorination (Gibbs Free Energy and Equilibrium Constant)

To evaluate the thermodynamic feasibility of the chlorination roasting reaction, the standard Gibbs free energy change (ΔG°) and equilibrium constant (K) were assessed using the following relations stated in Equations (4) and (5):

ΔG°_rxn_ = ΔH°_rxn_ − TΔS°_rxn_ (4)

ΔG = ΔG° + RTlnK (5)

**Figure 15:**
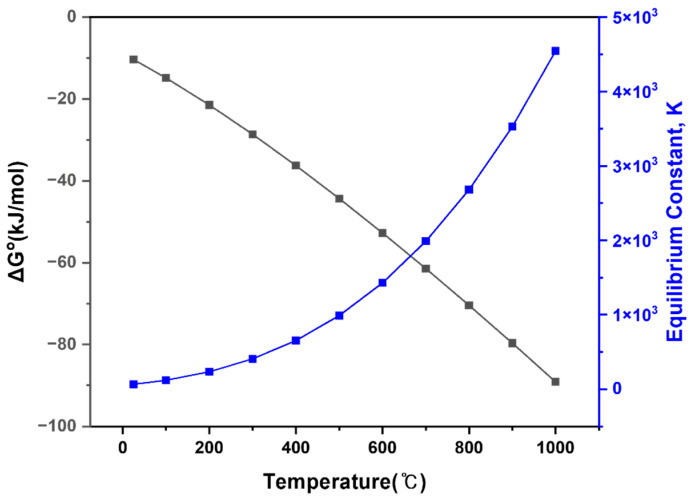
Temperature dependence of the standard Gibbs free energy change (ΔG°) and equilibrium constant (K) for the chlorination roasting reaction.

As shown in Figure 15, ΔG° remained negative over the investigated temperature range, indicating that the chlorination reaction is thermodynamically spontaneous. In addition, the equilibrium constant increased markedly with increasing temperature, suggesting that higher temperatures favor product formation and drive the reaction toward completion.

#### 3.2.2. Chlorination Roasting of H_2_-Reduced Products with CaCl_2_

This section evaluates CaCl_2_-assisted chlorination as a route to convert Li_3_PO_4_ into water-soluble LiCl and identifies the temperature window that maximizes LiCl recovery while limiting phosphate-derived byproduct formation. Chlorination roasting was conducted by reacting the H_2_-reduced product with calcium chloride (CaCl_2_), followed by phase identification using X-ray diffraction (XRD). CaCl_2_ was selected as the chlorinating agent due to its high ionic character and strong affinity for forming stable chloride products under high-temperature conditions. The chlorination experiments were performed at 700 °C, 800 °C, 900 °C, and 1000 °C for a fixed holding time of 3 h. The experimental conditions and sample inputs are summarized in Table 10. The solid mixture was placed in a magnesia crucible, which had been previously confirmed to be non-reactive under the relevant reducing/chlorinating environments.

**Figure 16:**
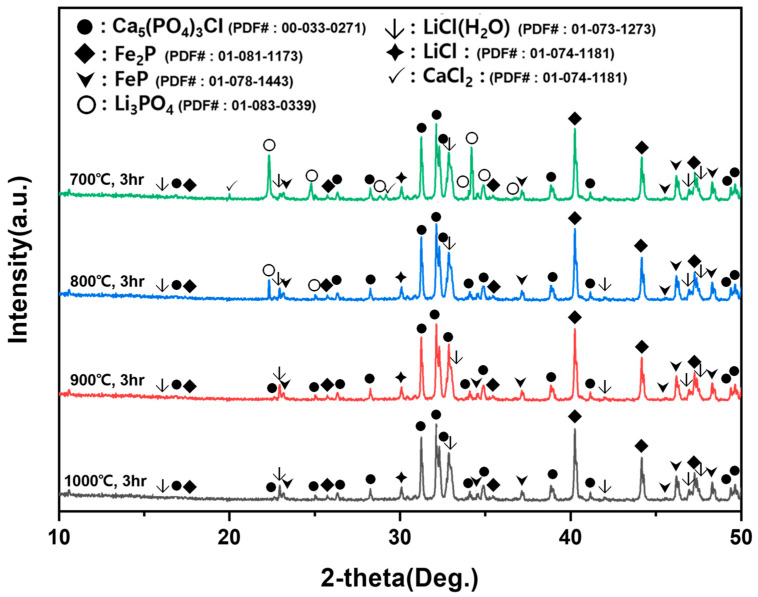
XRD patterns of chlorination-roasted products obtained at 700–1000 °C for 3 h. Major phases include LiCl, chlorapatite (Ca_5_(PO_4_)_3_Cl), FeP, and Fe_2_P; residual CaCl_2_ and/or phosphate-related phases may remain at lower temperatures (e.g., 700 °C) due to incomplete melting/reaction of CaCl_2_.

XRD patterns of chlorination products obtained at different temperatures are presented in Figure 16. Across all conditions, iron phosphides (FeP and Fe_2_P) persisted, indicating that these phases did not participate in the chlorination reaction and remained largely inert under the applied conditions. At 700 °C, unreacted CaCl_2_ and/or phosphate-containing phases were still detectable, which is attributable to insufficient melting of CaCl_2_ (melting point: 772 °C) and limited interfacial contact between reactants. In contrast, from 900 °C and above, the disappearance of unreacted reactants and stabilization of product phases suggested that chlorination proceeded effectively and reproducibly. Notably, XRD revealed the formation of LiCl together with chlorapatite (Ca_5_(PO_4_)_3_Cl) rather than solely calcium phosphate (Ca_3_(PO_4_)_2_). This indicates that secondary reactions occurred between CaCl_2_ and phosphate phases, leading to the stabilization of chlorapatite under the present experimental conditions.

Stabilization of chlorapatite and secondary reactions. The appearance of chlorapatite (Ca_5_(PO_4_)_3_Cl) instead of the initially expected Ca_3_(PO_4_)_2_ suggests that the Ca–P–O framework generated during chlorination can incorporate chloride and reorganize into an apatite-type structure under CaCl_2_-rich high-temperature conditions. Above the melting point of CaCl_2_ (772 °C), molten CaCl_2_ acts as an ionic flux that enhances mass transport and promotes solid–liquid reactions, thereby accelerating the conversion of phosphate-bearing solids toward thermodynamically stable Ca–P–Cl phases. In this environment, Ca_3_(PO_4_)_2_ formed locally (or transiently) can undergo chlorination/anion-exchange with CaCl_2_ to yield chlorinated calcium phosphate species (chlorophosphate/apatite-related intermediates), which subsequently stabilize as chlorapatite upon equilibration and cooling. Representative secondary pathways can be expressed as follows:

3Ca_3_(PO_4_)_2_ (s) + CaCl_2_ (s,l) → 2Ca_5_(PO_4_)_3_Cl (s) (6)

Ca_3_(PO_4_)_2_ (s) + CaCl_2_ (s,l) → 2Ca_2_PO_4_Cl (s) (7)

3Ca_2_PO_4_Cl (s) → Ca_5_(PO_4_)_3_Cl (s) + CaCl_2_ (L) (8)

where Equations (8) and (9) describe a plausible intermediate (Ca_2_PO_4_Cl) route in Ca–P–Cl systems at elevated temperatures, and the regeneration of CaCl_2_ (L) in Equation (9) is consistent with chlorapatite stabilization during thermal treatment/cooling.

The formation of chlorapatite can influence LiCl recovery in two ways. First, it can reduce the effective availability/activity of chloride (Cl^−^) for LiCl formation because chloride becomes incorporated into Ca–P–Cl solids. Second, because LiCl is molten at the reaction temperatures used here (m.p. 605 °C), molten LiCl may partially wet, infiltrate, or become occluded within the evolving phosphate matrix during reaction and subsequent cooling/solidification, which can hinder dissolution during water leaching and lower the apparent Li recovery. Therefore, suppressing chlorapatite formation (e.g., by optimizing CaCl_2_ dosage, temperature/time, and melt fraction) and minimizing LiCl retention in phosphate-derived solids are key to improving practical LiCl yield and lithium recovery.

Based on the temperature-dependent chlorination behavior, the optimal chlorination temperature was determined by subsequent water-leaching performance and lithium content in the recovered solution.

#### 3.2.3. Water Leaching and Solid–Liquid Separation

**Figure 17:**
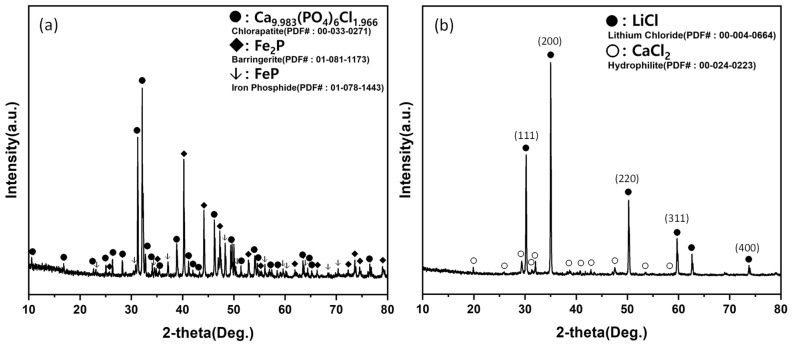
XRD patterns after water leaching and filtration: (**a**) dried residue and (**b**) dried filtrate.

This section demonstrates the selective separation of LiCl from insoluble residues by water leaching and vacuum filtration and explains the temperature-dependent lithium recovery behavior. LiCl was recovered by exploiting its high solubility in water. After chlorination roasting, the products were subjected to water leaching using a magnetic stirrer, followed by vacuum filtration to separate the LiCl-containing filtrate from insoluble residues. Residual Ca^2+^ in the filtrate was removed by cation-exchange resin treatment, and the purified filtrate was dried/dehydrated to obtain LiCl powder. Water leaching was performed to selectively dissolve water-soluble LiCl from the chlorination-roasted products while retaining insoluble phases (e.g., chlorapatite and iron phosphides) in the residue. Leaching was conducted using deionized water at a solid-to-liquid ratio of 1:10, with stirring at 300 rpm and 25 °C for 24 h. After leaching, the slurry was vacuum-filtered to separate the solid residue and filtrate, and both fractions were dried prior to characterization. The XRD patterns of the dried residue and dried filtrate are shown in Figure 17. The residue predominantly contained insoluble phases such as Ca_5_(PO_4_)_3_Cl and iron phosphides, whereas the dried filtrate exhibited diffraction peaks consistent with LiCl, confirming selective extraction of LiCl into the aqueous phase. ICP–OES analysis of the dried filtrate revealed that the lithium content decreased from 15.7 wt.% (900 °C) to 13.21 wt.% (1000 °C), while the calcium content increased from 4.32 wt.% (900 °C) to 5.86 wt.% (1000 °C). This trend indicates that excessive temperature may reduce the effective recovery of water-soluble LiCl. A plausible explanation is that LiCl (melting point: 605 °C) exists as a molten salt at both 900 °C and 1000 °C. At higher temperatures, it may partially infiltrate or become occluded within the phosphate matrix (e.g., chlorapatite) during cooling and solidification. Such encapsulation can hinder dissolution during water leaching, thereby decreasing the apparent lithium recovery in the filtrate. Based on the filtrate lithium content, 900 °C was selected as the optimal chlorination temperature for subsequent purification steps. The optimal chlorination temperature was selected based on the higher Li content and lower Ca carryover in the filtrate-derived product, indicating more effective liberation of water-soluble LiCl during leaching. Because Ca^2+^ remained in the filtrate at the wt.% level, an ion-exchange purification step was subsequently applied to remove Ca^2+^ prior to final LiCl recovery (Section 3.2.4).

#### 3.2.4. Ca^2+^ Removal by Cation-Exchange Resin and Recovery of LiCl Powder

**Figure 18:**
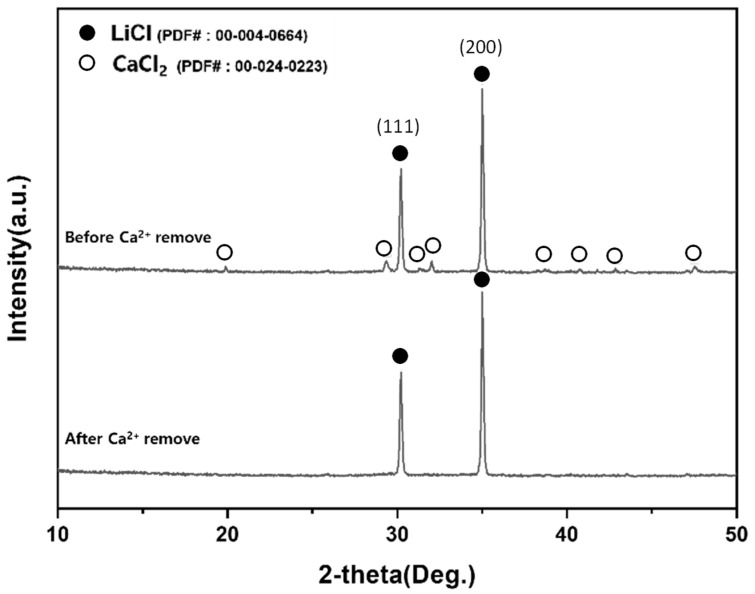
XRD comparison of the dried filtrate before and after Ca^2+^ removal by ion-exchange resin.

This section quantifies the effectiveness of ion exchange in removing Ca^2+^ impurities from the LiCl-containing filtrate, thereby improving product purity. Following water leaching, Ca^2+^ remained in the LiCl-containing filtrate as an impurity originating from CaCl_2_ and calcium-bearing phosphate species. To improve the purity of the recovered LiCl, Ca^2+^ was removed using a strong-acid cation-exchange resin (H-form). Based on the measured Ca content in the filtrate and the resin exchange capacity (≥2.4 eq L^−1^; resin density: 815 g L^−1^), 1.56 g of resin was added to the filtrate, and the suspension was stirred at 300 rpm for 1 h to promote ion exchange and Ca^2+^ uptake. The resin was then separated by vacuum filtration, and the purified LiCl solution was dried to obtain LiCl powder. The phase changes before and after resin treatment were evaluated by XRD (Figure 18), confirming the reduction in calcium-containing species after purification.

The purified solution was then dried to obtain LiCl powder. The chemical composition of the recovered product is summarized in Table 11, showing a substantial decrease in Ca content after ion exchange. For dehydration control during drying, the filtrate was first dried at 120 °C for 24 h, followed by an additional heat treatment at 400 °C for 5 h sto remove residual hydration water while minimizing LiCl volatilization.

#### 3.2.5. LiCl Powder Lithium Mass Balance and Recovery

Lithium recovery was calculated based on the lithium mass in the final LiCl powder relative to the lithium mass in the initial LiFePO_4_ feed, as stated in Equation (9):

(9) $$\mathrm{Li}\;\mathrm{recovery}\;(\%)\;=\;\frac{m_{Li\;in\;recovered\;LiCl}}{m_{Li\;in\;raw\;material}}\times 100$$

**Figure 19:**
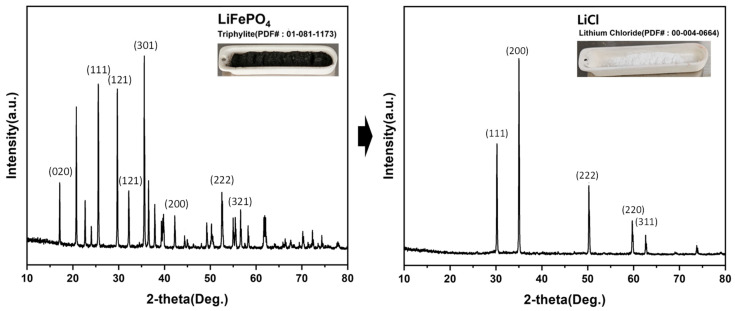
XRD patterns of the initial LiFePO_4_ powder and the recovered LiCl powder, confirming phase transformation and LiCl formation.

The sample mass and lithium content at each major step are summarized in Table 12. Starting from 10.000 g of LiFePO_4_, 2.009 g of LiCl powder was obtained. Based on the measured lithium contents, 0.327 g of lithium was recovered from an initial 0.456 g, corresponding to a lithium recovery of 71.7%. To visually corroborate the overall chemical conversion achieved through the proposed route (hydrogen reduction → chlorination roasting → water leaching/purification), an XRD comparison between the initial LiFePO_4_ feed and the final recovered LiCl powder is presented in Figure 19. The disappearance of the characteristic olivine LiFePO_4_ reflections and the emergence of diffraction peaks corresponding to LiCl confirm the successful phase transformation of lithium species into LiCl. This phase verification, together with the lithium mass balance (Table 12), supports the conclusion that lithium was effectively recovered as LiCl, with a recovery of 71.7%.

Although SEM/TEM images are not provided, the before/after transformation is supported by the stepwise XRD evidence (raw LFP → Li_3_PO_4_/Fe phosphides → LiCl + phosphate phases → recovered LiCl), together with ICP–OES-based composition tracking and the lithium mass balance.

To clarify the novelty and positioning of the present route relative to previously reported LFP recycling strategies, representative literature is compared with this work in Table 13, focusing on process concept, recovered lithium form, purification approach, and reported recovery/extraction metrics (as defined in each reference).

As summarized in Table 13, this work differs from prior chlorination-based approaches by incorporating hydrogen-reduction-driven phase separation prior to chlorination and by applying ion-exchange purification to reduce Ca^2+^ impurity in the LiCl stream, which is important for downstream lithium chemical synthesis.

## 4. Conclusions

This study investigated an environmentally oriented recycling route for recovering lithium from LiFePO_4_ (LFP) cathode active materials, targeting the production of lithium chloride (LiCl) as a reusable lithium intermediate. Unlike conventional routes that may involve extensive chemical consumption and waste generation, the proposed approach combines hydrogen reduction with CaCl_2_-assisted chlorination followed by aqueous separation, aiming to simplify the separation chemistry while maintaining practical recovery.

Hydrogen reduction achieved complete decomposition of LiFePO_4_ and phase separation into Li_3_PO_4_, FeP, and Fe_2_P, and the observed mass-retention ratio closely matched the theoretical value (~72.96%) associated with oxygen removal as H_2_O(g), leading to the identification of 900 °C for 1 h as the optimal reduction condition. Subsequently, Li_3_PO_4_ in the reduced product was converted to water-soluble LiCl via CaCl_2_-assisted chlorination while FeP and Fe_2_P remained largely inert; LiCl was then selectively recovered into the filtrate by water leaching and vacuum filtration, yielding an overall lithium recovery of 71.7% based on the initial LiFePO_4_ feed and final LiCl product. XRD confirmed the formation of chlorapatite (Ca_5_(PO_4_)_3_Cl) rather than the expected Ca_3_(PO_4_)_2_, indicating secondary reactions that likely consume chloride (Cl^−^) and reduce the effective LiCl yield. Meanwhile, additional losses may arise from the retention/occlusion of molten LiCl within the phosphate matrix during cooling and solidification, which hinders dissolution during leaching. The recovered LiCl powder is suitable as a recyclable lithium intermediate and can be subsequently converted to Li_2_CO_3_ and LiOH, providing a practical route to reintegrate lithium into battery-material supply chains. Future work should therefore focus on suppressing chlorapatite formation and minimizing LiCl retention, for example, by optimizing temperature (near or below the LiCl melting point where feasible) and/or applying a physical separation approach (e.g., tapping/draining) to recover molten LiCl prior to solidification.

## Figures and Tables

**Table 1:** Chemical composition of the LiFePO_4_ powder determined by bulk elemental analysis (sample mass: 10.0 g), compared with theoretical values.

Sample Weight (g)		
10.0		
Element	Theoretical wt.%	Analyzed wt.%
Li	4.40	4.56
Fe	35.4	35.1
P	19.6	18.4

**Table 2:** Hydrogen content of LiFePO_4_ powder before and after calcination pretreatment measured by elemental analysis (EA).

Element	Raw Material (wt.%)	Calcined LFP (wt.%)
H	0.017	-

**Table 3:** Equilibrium phase distribution of MgO (unit: mol) in the MgO crucible case under hydrogen-reduction conditions, showing Total MgO = 5.0000 mol over 300–900 °C (unit: mol).

	300 °C	400 °C	500 °C	600 °C	700 °C	800 °C	900 °C
MgO	3.24100	3.09050	2.97070	2.87290	2.79140	2.72210	2.66240
MgO(M)	1.75900	1.90950	2.02930	2.12710	2.20860	2.27790	2.33760
Total MgO	5.00000	5.00000	5.00000	5.00000	5.00000	5.00000	5.00000

**Table 4:** Equilibrium phase distribution of Al_2_O_3_ (unit: mol) in the Al_2_O_3_ crucible case under hydrogen-reduction conditions, showing Total Al_2_O_3_ < 5.0000 mol over 300–900 °C, indicating alumina participation in side reactions (unit: mol).

	300 °C	400 °C	500 °C	600 °C	700 °C	800 °C	900 °C
Al_2_O_3_(C)	1.91080	1.85730	1.81200	1.75090	1.66090	1.54440	1.41280
Al_2_O_3_	1.82340	1.75890	1.71500	1.65800	1.57440	1.46590	1.34290
Al_2_O_3_(D)	0.28459	0.38192	0.47762	0.56278	0.62835	0.66995	0.68888
Al_2_O_3_(K)	0.16561	0.25114	0.34310	0.43206	0.50785	0.56396	0.59923
Al_2_O_3_(G)	0.04775	0.08801	0.13989	0.19903	0.25886	0.31327	0.35854
Total Al_2_O_3_	4.23215	4.33727	4.48761	4.60277	4.63036	4.55748	4.40235

**Table 5:** Solubility of LiCl in water as a function of temperature.

Temperature (°C)	Solubility in Water (LiCl)
0	68.29
10	74.48
25	84.25
40	88.7
100	123.44

**Table 6:** Calculated equilibrium composition for LiCl·H_2_O thermal decomposition at 200–800 °C (unit: mol).

	200 °C	300 °C	400 °C	500 °C	600 °C	700 °C	800 °C
H_2_O (g)	0.78663	0.98152	0.99706	0.99925	0.99973	0.99988	0.99989
LiCl	0.78663	0.98152	0.99706	0.99924	0.99965	0.9991	0.99527
LiCl·H_2_O	0.21337	0.01848	0.00294	0.00075	0.00026	0.00009	0.00003
H_2_O	0	0	0	0	0	0	0

**Table 7:** Hydrogen-reduction conditions and mass-change results for LiFePO_4_ (H_2_ flow rate: 300 cm^3^ min^−1^; initial mass: 10.0 g; heating rate: 5 °C min^−1^).

Sample	Temperature (°C)	Reaction Time (h)	H_2_ Flow Rate (cm^3^ min^−1^)	Initial Mass (g)	Product Mass (g)	Mass-Retention Ratio (%)
#1	700	1	300	10	9.685	96.85
#2	700	2	300	10	8.638	86.38
#3	700	3	300	10	8.513	85.13
#4	800	1	300	10	7.667	76.67
#5	800	2	300	10	7.606	76.06
#6	800	3	300	10	7.518	75.18
#7	900	1	300	10	7.292	72.92
#8	900	2	300	10	7.274	72.74
#9	900	3	300	10	7.256	72.56

# denotes the sample number.

**Table 8:** Stoichiometric calculation of theoretical mass change associated with oxygen removal during hydrogen reduction of LiFePO_4_ based on Equation (3) (basis: 10.0 g of LiFePO_4_).

Basis (10.0 g LiFePO_4_)	Li	Fe	P	O	Total
Amount in raw material (mol)	0.063388	0.063388	0.063388	0.253554	0.443719
Amount in raw material (g)	0.439979	3.539929	1.963382	4.05671	10
Amount in reduced product (mol)	0.063388	0.063388	0.063388	0.084518	0.274683
Amount in reduced product (g)	0.439979	3.539929	1.963382	1.352237	7.295527
Oxygen removed (mol)	—	—	—	0.169036	—
Oxygen removed (g)	—	—	—	2.704473	—
Theoretical mass-retention ratio (%)					72.96

**Table 9:** Chemical composition of the hydrogen-reduction product obtained under the selected condition (900 °C, 1 h), compared with theoretical values (sample mass: 7.29 g).

Sample Weight (g)		
7.29		
Element	Theoretical wt.%	Analyzed wt.%
Li	6.03	6.11
Fe	48.5	47.1
P	26.9	25.1

**Table 10:** Experimental conditions for chlorination roasting of the H_2_-reduced product with CaCl_2_ (Ar: 300 cm^3^ min^−1^; holding time: 3 h).

Sample	H_2_ Reduction		Raw Mat.	Sample Input (g)	
	Temp.	Reaction Time	Ar (cc/min)	H_2_ Reduction Product	CaCl_2_
	(°C)	(h)			
#1	700	3	300	7.292	3.562
#2	800				
#3	900				
#4	1000				

# denotes the sample number.

**Table 11:** Chemical composition of the dried product after Ca^2+^ removal (ICP-OES, wt.%).

Sample Weight (g)	
2.009	
Element	Analyzed wt.%
Li	16.3
Ca	0.17

**Table 12:** Lithium content and mass balance by experimental step and calculated lithium recovery.

Experimental Step	Sample wt.%	Li Content	Li wt.%	Recovery Rate
	(g)	(%)	(g)	(%)
Raw material (LiFePO_4_)	10	4.56	0.456	-
After H_2_ reduction	7.292	6.11	0.446	-
Recovered LiCl (powder)	2.009	16.3	0.327	71.70%

**Table 13:** Comparison of representative LFP recycling routes and key outcomes reported in the literature versus this work.

Study	Route Type/ Key Steps	Main Recovered Li Form	Purification (Key)	Reported Li Recovery/Extraction (as Defined in Each Reference)	Notes/Limitation
Barbosa de Mattos et al. [2]	Review (hydrometallurgy + hybrid/thermal)	Li_2_CO_3_/LiOH/others (varies)	Various	–	Highlights reagent/wastewater burden and flowsheet complexity
Zhao et al. [3]	Review (direct regeneration)	Re-lithiated LFP	–	–	Feed variability and scale-up challenges
Cao et al. [12]	CaCl_2_ chlorination roasting of LFP	LiCl (leaching)	Limited/depends	(reported in ref.)	Phosphate byproducts may form; condition-sensitive
Chang et al. [13]	CaCl_2_-assisted roasting (LFP)	Li salts (leaching)	Fluorine stabilization focus	(reported in ref.)	Emphasizes stabilization/impurity control
Kim et al. [9]	Chlorination roasting + leaching (spent LIBs)	LiCl/Li salts	Process-dependent	(reported in ref.)	Chlorination can benefit leaching; impurity management important
This work	H_2_ reduction → Li_3_PO_4_ + FeP/Fe_2_P (phase separation) → CaCl_2_ chlorination → water leaching/vacuum filtration → Ca^2+^ ion exchange → drying/dehydration	LiCl (powder)	Ca^2+^ removed by strong-acid cation exchange (H-form)	71.70%	Addresses Ca impurity in LiCl stream; discusses chlorapatite formation and LiCl retention mechanisms

## Data Availability

The original contributions presented in this study are included in the article. Further inquiries can be directed to the corresponding author.
